# Supplementary material for: A paired-end whole-genome sequencing approach enables comprehensive characterization of transgene integration in rice
Source: Commun Biol. 2022 Jul 5;5:667. doi: 10.1038/s42003-022-03608-1 (PMC9256713; doi:10.1038/s42003-022-03608-1)
Supplement: Supplementary file 2 — Supplementary Information [file 42003_2022_3608_MOESM2_ESM.pdf]

Supplementary Information

A paired-end whole-genome sequencing approach enables comprehensive characterization of transgene integration in rice

Wenting Xu[1], *Hanwen Zhang*[1], *Yuchen Zhang*[1], *Ping Shen*[2], *Xiang Li*[3], *Rong Li*[1], *Litao Yang*[1]*

[1] National Center for the Molecular Characterization of Genetically Modified Organisms, Joint International Research Laboratory of Metabolic and Developmental Sciences, School of Life Sciences and Biotechnology, Shanghai Jiao Tong University, Shanghai 200240, China.

[2] Development Center of Science and Technology, Ministry of Agriculture of People's Republic of China, Beijing 100025, China.

[3] Technical Center for Animal, Plant and Food Inspection and Quarantine of Shanghai Customs, Shanghai 200135, China.

*To whom correspondence should be addressed: yyllttt@sjtu.edu.cn.

Supplementary Tables

Supplementary Table 1. The detail information of seven sporadic candidate paired-end reads in G281 rice

Read pair name	Chromosome	Mapped location	Sequence information
FCC3208ACXX:2:1304:1671:88133#	2	NC_029257.1:25203045	GCTCCACCTTGTCCATGCGGGACACCACGGCGTGGTTCGGGGCCATGGCGAGGTGGCAGGAGCGGGCCTCGGTCACCGGCTTGCGCTTGCCGTCGAGGCA
		NC_029257.1:25203045	TTCTTTACGAATACGGAGGGAGTAGCTATAAGTTTATTTTTTATAGCATGATGGTGTATACACGTGGAGTATGTACGTACCTGAGGGAGGTGAGGTCGCC
FCC3208ACXX:2:1311:20752:71131#	2	NC_029257.1:9581053	AGCCAAAAATTTAGGGATGAGCACCAAAAGATCCACCAATTTAGACAAGGAGATATCGTTGCACTGCCAGCTGGTGTTGCGCATTGGTTCTACAATGAAG
		NC_029257.1:9586959	AGTTTAAGGGCATTCTTTACCCGTATGATCTCTCCTCTTTGGTCGTTTTGGCCTTGTAGCCTCTTTGCTACCAATGCATTGACGCCTAAGGCCTCACTTA
FCC3208ACXX:2:1310:6662:74129#	4	NC_029259.1:817940	AGATTGAGCGAGAGCGAGAGATTAAGGGAATGAATGAGCGGATCCTTTATTGATAACAGTGTTACAATATATAGGAGGGAGAAAGGCGGCAACCGAAGGC
		NC_029266.1:12123362	GTTAGCTCACTCATTAGGCACCCCAGGCTTTACACTTTATGCTTCCGGCTCGTATGTTGTGTGGAATTGTGAGCGGATAACAATTTCACACAGGAAACAG
FCC3208ACXX:2:1213:2984:21437#	6	NC_029261.1:13168191	AAACTTGTTGGGTGCCATCCAGGAGCCAGCACCGCACACAACATGGAGGCCACCTGTGGCATGCGGTCTCACATGTATATGTTTGACCCAGGGTGCACAG

FCC3208ACXX:2:2211:6613:28129#	8	NC_029265.1:13499802	CAAACGTGACTTATGGGTTTATGTTCTAACATTTAGCTGAAGATACTTATGTAGATGAGTAGGAATGAATAGGTTGAGAGAGGCATGATATGATGCATGA
		NC_029263.1:11331616	TTGTTCCAGCTTGTTATATGGAGTTTATTAGAGTTAAAGCCTTTTCTTTTCCGTTGGTCGTGTAGTCGCCGCAGCTTCGGAAGATCCGCTCTTCGTGGAA
		NC_029265.1:13499328	GCAAACTGTGAAGAAAACTATGGGGCGATTTATGGATGCCATGTTGTTGTAGGACTAATGAACTGAATGCTTTTTGTGAGAAACAATGAGAAATCATCGT
FCC3208ACXX:3:2206:20723:10696#	11	NC_029257.1:8459960	TCACCATCATTGTAGAACCAATGTGCAACACCAGCTGGGAGTGCAACAATGTCTCCTTGCCTAAATTGATGAATCTTTTGGTGCTCATCTCTAAACTTTT
		NC_029266.1:1798829	CAGATAACAGTTCATTCCCGGATTCATGAAATGGCCCAAGAAGCCGGTTGGTTGGTTGGTTGGAGGCACTACGTAGAGGCCTCATCGCCACCAAATGCAT
FCC3208ACXX:3:1107:12372:67498#	12	NC_011033.1:191960	CCCATGCTTATATAATCCAATCAATTGCACCATGATTAATCCACTAGACAAAAGGGAAACACAATCACTACATTAACCGAGCTATATTAGCCGCGCTTTC
		NC_011033.1:191960	GCAAGTGCGGCCTCGTGCCGGTGCTCGCCGAGAACTACAAGTCCCAGCAGTCCTCCGACCCGGACCCGAACTGCGTGGACCGCCCGGTGGAGGGCTACCT

Supplementary Table 2. Estimation of the copy number of transgenes in artificial cisgenesis rice using PE-WGS

Gene	In silico cisgenic line			WT			Copy number
	ADT_{gm}	D_{gm}	R_{gm}	ADT_{wt}	D_{wt}	R_{wt}	
Gt1 (2052 bp)	67.35	29.28	0.989	37.83	29.28	0.989	1.17
SPS	25.14	29.28	0.989	25.14	29.28	0.989	/

Supplementary Table 3. Primer pairs designed for transgene insertion locus validation

**Primer name**	**Sequences (5’->3’)**	Amplicon length (bp)
Chr3-F1	CTTTCAACGTGACCGCTGAC	614
Chr3-R1	TTCGTGAGCTCAGCTTAGCC	
Chr3-F2	CGTCTAGCTGAAGGCCCATT	374
Chr3-R2	TTCGTGAGCTCAGCTTAGCC	
Chr2-F1	CTGAGGGAGGTGAGGTCGCC	
Chr2-R1	CGCTCCTGCCACCTCGCCAT	
Chr2-F2	AGAGGAGAGATCATACGGGT	
Chr2-R2	ATCTTTTGGTGCTCATCCCT	
Chr4-F	TAAGGGAATGAATGAGCGGA	
Chr4-R	AAAGTGTAAAGCCTGGGGTG	
Chr6-F	GGTGCCATCCAGGAGCCAGC	
Chr6-R	ACATAAACCCATAAGTCACG	
Chr8-F	GCCTTTTCTTTTCCGTTGGT	
Chr8-R	TCGCCCCATAGTTTTCTTCA	
Chr11-F	CAGATAACAGTTCATTCCCG	
Chr11-R	TGATGAATCTTTTGGTGCTC	
Chr12-F	CAAAAGGGAAACACAATCAC	
Chr12-R	CTACAAGTCCCAGCAGTCCT	

Supplementary Table 4. Primers and probes used for ddPCR analysis

**Primer name**	**Sequences(5’->3’)**	Amplicon length (bp)
G281-G6-EPSPS-F	TACGCCGAGGGCAAGACC	
G281-G6-EPSPS-R	CTCCACGCTGTAGCCGAAG	90bp
G281-G6-EPSPS-P	FAM-CCTCAGCATCCTCTCGGTGTGGTCC-BHQ1	
G281-HLF-F	CTCCTCTTCAACGACAAC	
G281-HLF-R	GAGCACTTCTTGAGGTTG	113bp
G281-HLF-P	FAM-CTCCACGGCAAGACCACCTA-BHQ1	
Rice-SPS-F	TTGCGCCTGAACGGATAT	
Rice-SPS-R	CGGTTGATCTTTTCGGGATG	81bp
Rice-SPS-P	FAM-TCCGAGCCGTCCGTGCGTC-BHQ1	

Supplementary Figures

Supplementary Figure 1. Conventional PCR amplification results for the seven sporadic candidate paired-end reads in G281 rice. Lane M, DL2000 marker; Lane GM, G281; Lane WT, Xiushui 110; Lane NTC, no template control.

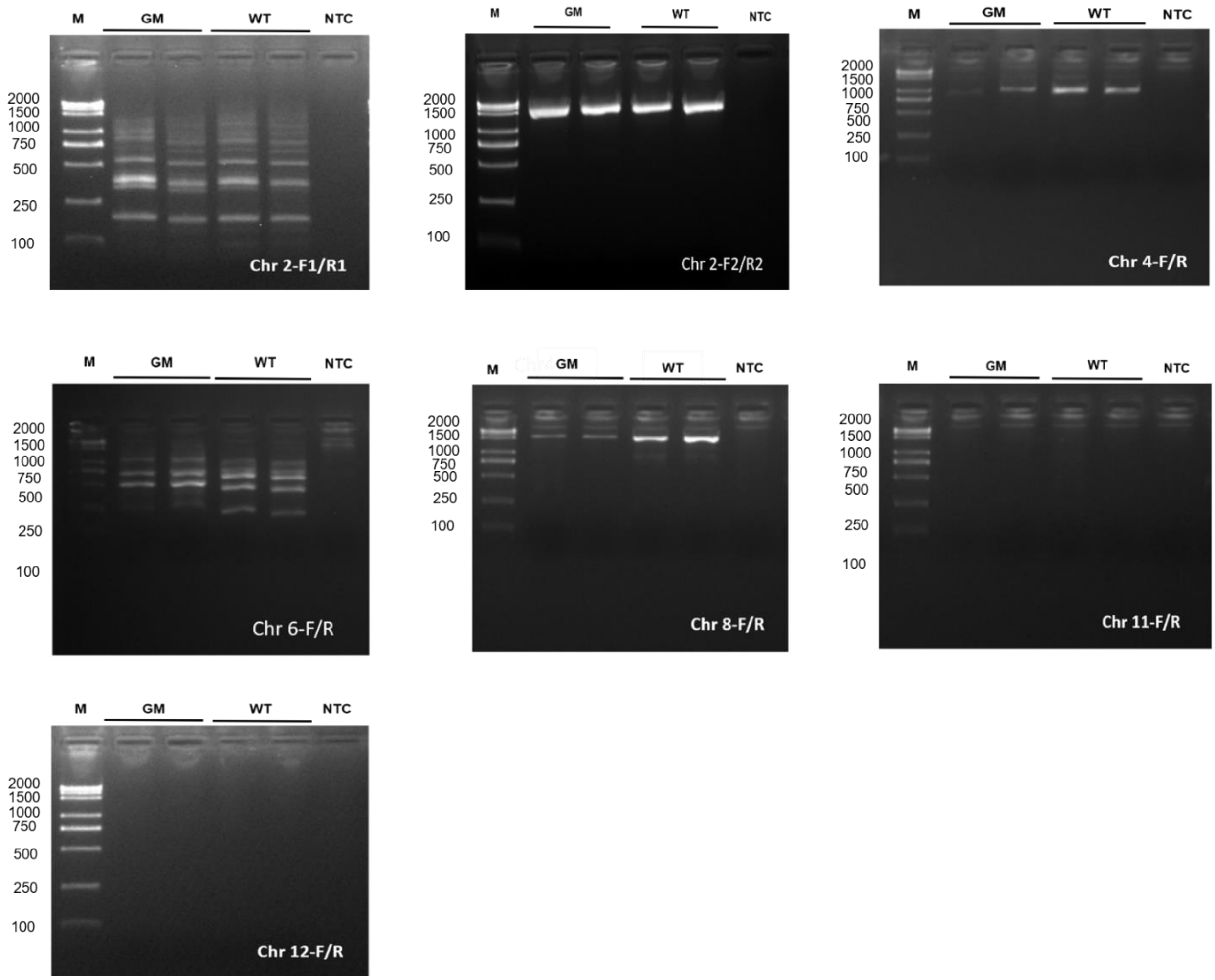

Supplementary Figure 2. IGV view of seven sporadic read pairs in rice reference genome.

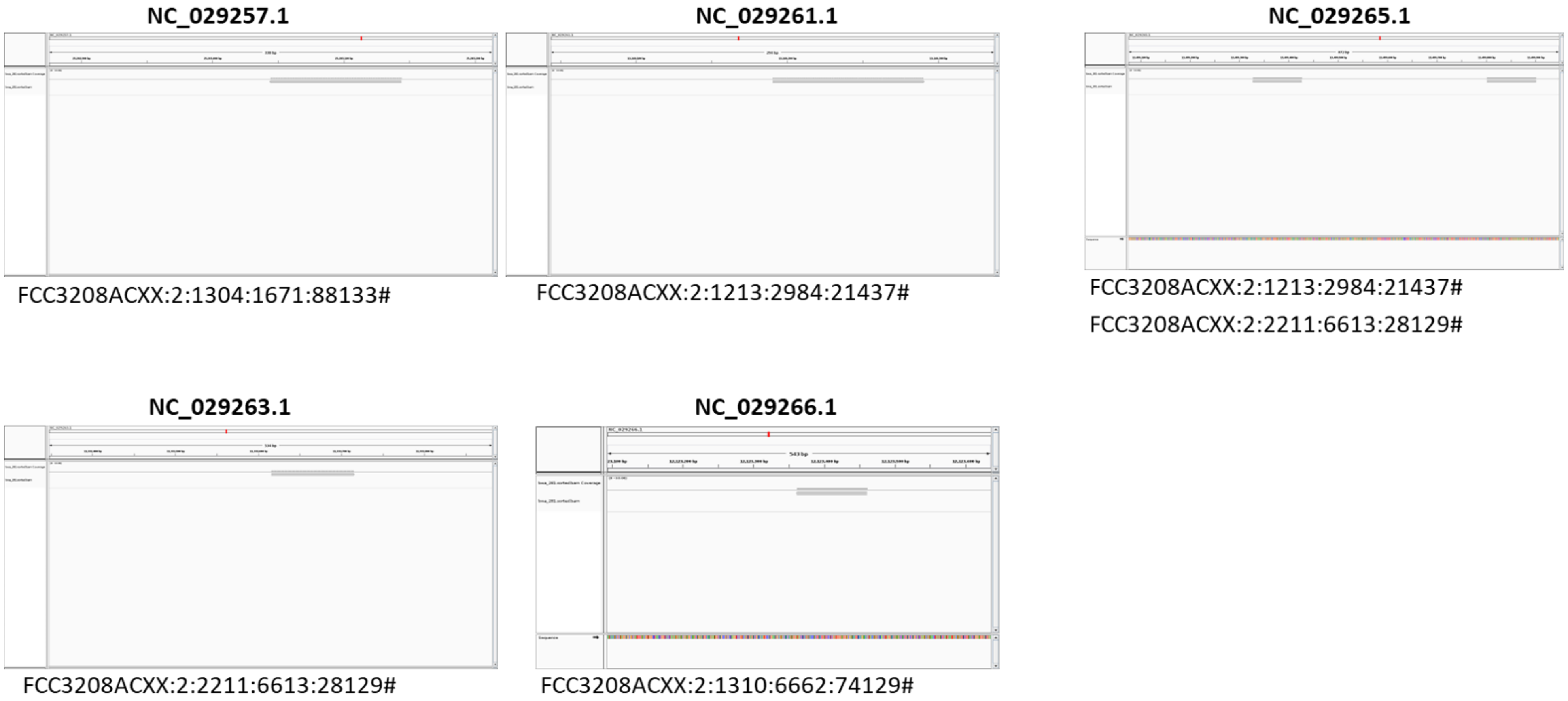

Supplementary Figure 3. Demo diagram of T-DNAs and its insertion in rice genome in transgenesis and cisgenesis/intragenesis.

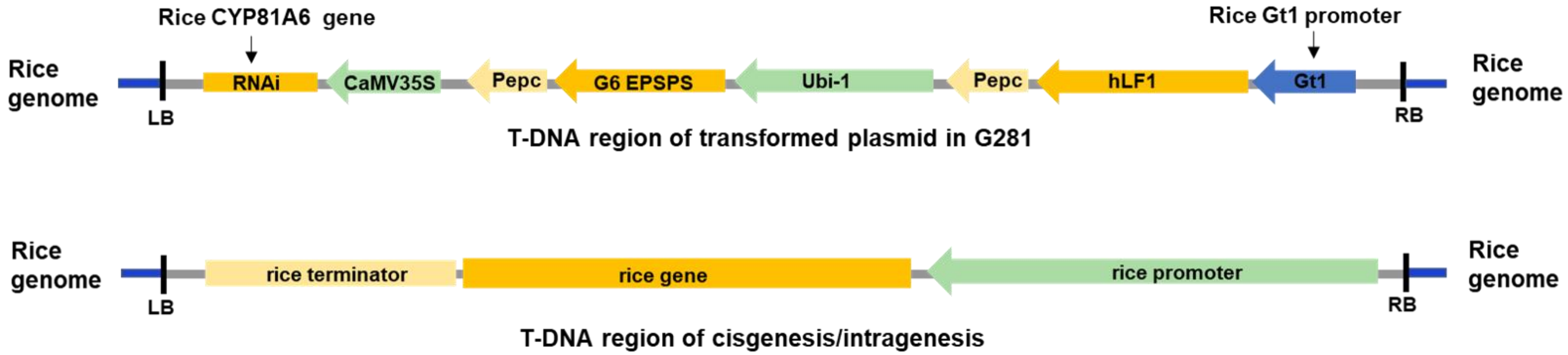

Supplementary Figure 4. The *Gt1* gene integration site and its flanking sequence from PE-WGS in artificial cisgenesis line. (A) IGV view of 116 candidate read pairs crossing the insertion site of chromosome 5. The red line indicates the cisgene insertion site in the rice genome. (B) Location of the cisgene integration site according to the assembled contigs from candidate read pairs.

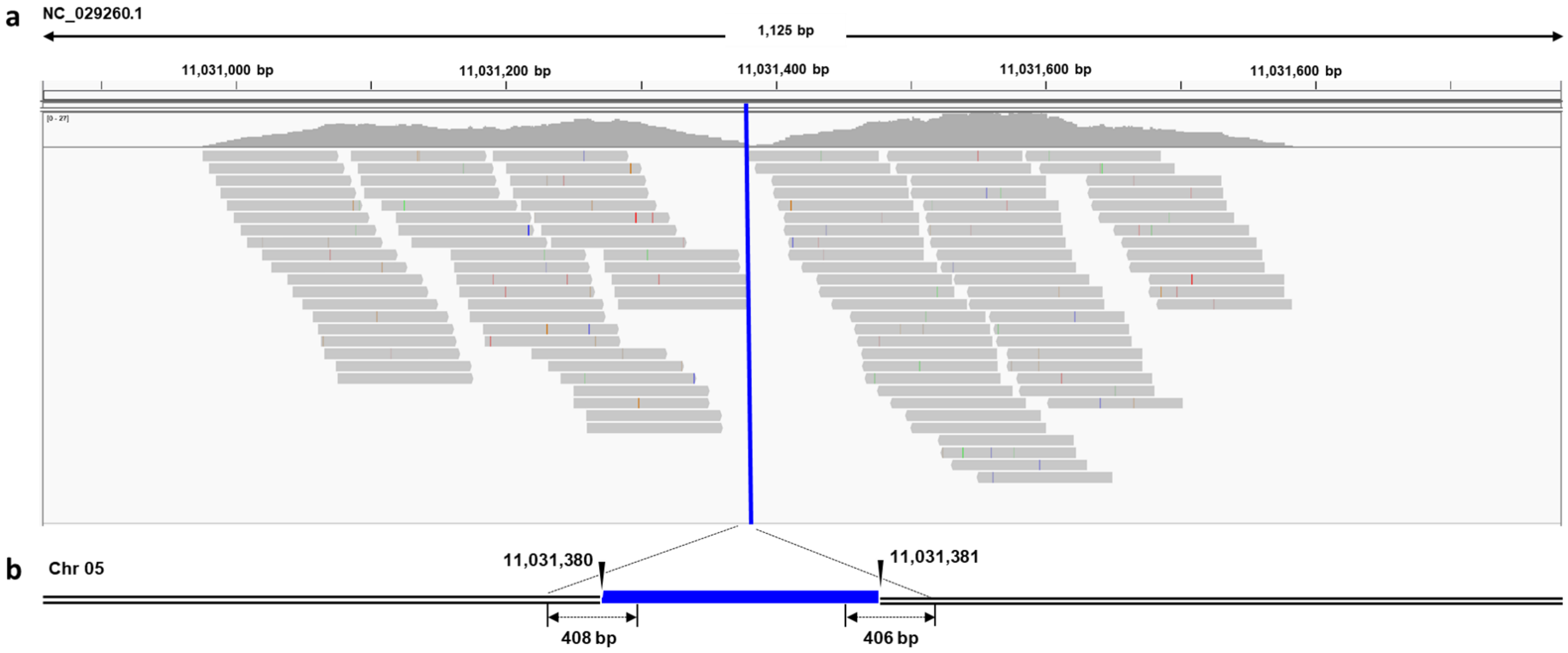

Supplementary Figure 5. The structure diagram of mimic *Gt1* gene insertion in artificial cisgenesis rice line.

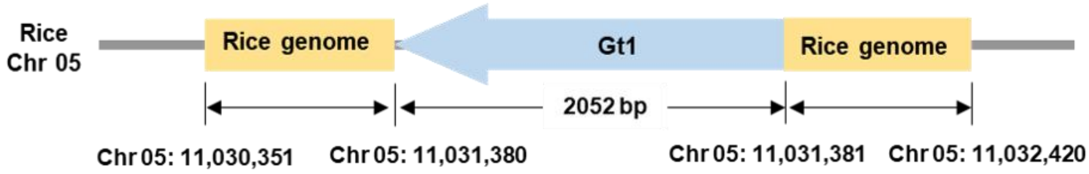

Supplementary Sequence Files

Supplementary Sequence File 1. Assembled contigs from the 69 candidate read pairs

>NODE_1_length_497_cov_10.398190
AAAATCATATCAATCCATTTTTTAAGTTATAGCTAATACTTAATTAATCATGCGCT
AATAAGTCACTCTGTTTTTCGTACTAGAGAGATTGTTTTGAACCAGCACTCAAGA
ACACAGCCTTAACCCAGCCAAATAATGCTACAACCTACCAGTCCACACCTCTTGT
AAAGCATTTGTTGCATGGAAAAGCTAAGATGACAGCAACCTGTTCAGGAAAACA
ACTGACAAGGTCATAGGGAGAGGGAGCTTTTGGAAAGGTGCCGTGCAGTTCAAA
CAATTAGTTAGCAGTAGGGTGTTGGTTTTTGCTCACAGCAATAAGAAGTTAATCA
TGGTGTAGGCAACCCAAATAAAACACCAAAATATGCACAAGGCAGTTTGTTGTAT
TCTGTAGTACAGACAAAACTAAAAGTAATGAAAGAAGATGTGGTGTTAGAAAAG
GAAACAATATCATGAGTAATGTGTGAGCATTATGGGACCACGAAATAAAAAAAG
AACAT

>NODE_2_length_478_cov_5.617021 (Confirmed 5' flanking sequence of the transgene insertion)
CCTTCCCATGCCGTCAGGCTCAGGCATCACCGCGTTCTCACAAGTCACACCGAGT
CCGCTGCCTTTCAACGTGACCGCTGACCGCTCGAGGCCAAGGGCGCGACCGCCGT
CTCCCTCCCCTGCACCACGGCCTCCCTCCCCAGCGTCGCTCCACGGTCAACGCTCC
CTACACCACCGATGTCCCTAACTCGATGTCGTATGCTACTGATGGTACTTGTCCTT
GCTGCTGCATTGCATTATTTGAGGCCAACGTGACCTGTAGGAGTTTGATCCTAAT
GCATACTTGATGTATACTTGTTGAAAATTGATTGCTTTATTGCTGAAGTGTTTACT
GCTGCTAGTATCACGAGCTGGCTCAAGCTTTGAAATGAGCCAAGCCAAACTTATA
TGTACTGAATTAACGCCGAATTAATTCGGGGGATCTGGATTTTAGTACTGGATTTT
GGTTTTAGGAATTAGAAATTTTATTGATAGAAGT

>NODE_3_length_470_cov_4.913253
CCTACAACAACATGGCATCCATAAATCGCCCCATAGTTTTCTTCACAGTTTGCTTG
TTCCTCTTGTGCGATGGCTCCCTAGCCCAGCAGCTATTAGGCCAGAGCACTAGTC
AATGGCAGAGTTCTCGTCGTGGAAGTCCGAGAGGATGTAGATTTGATAGGTTGCA
AGCATTTGAGCCAATTCGGAGTGTGAGGTCTCAAGCTGGCACAACTGAGTTCTTC
GATGTCTCTAATGAGTTGTTTCAATGTACCGGAGTATCTGTTGTCCGCCGAGTTAT
TGAACCTAGAGGCCTACTACTACCCCATTACACTAATGGTGCATCTCTAGTATAT
ATCATCCAAGGTTTGTGTAACAATTTAAGTGCATAATGAATTAATGATTGGCTGC
GATATTTACATTGCTTGTAATTAACATGCATGCCATACTTTCAGGGAGAGGTATA
ACAGGGCCAACTTTCCCAGGCTGTCCTG

>NODE_4_length_434_cov_3.295515 (Confirmed 3' flanking sequence of the transgene insertion)
AGGGAGACAAAAGTGCAAGGAAGAAAGAGAAGCATACCAACCAATAGCTTCAC
ATGAGCTGTACAGTGGAAACGCTTTACTTTGTATGCTCCAATGTTAATTTGCTTCC

TTCCTCCTTATCTACCATCGTTTCCTTGTATGCATCCATAGCTTTCAGGAAGTCAA
TATATCAAAAAAGAAAAAAAAAAAGCTTTGCGAAAAACTATCTTGGACTTAAATA
CCAGTCAGATGTCTTGATTTGTTCTTTGGCACAGTGTATTTATTTTGGTACAGTAT
ATTGTCGATGTGTAGACCTCGTCTAGCTGAAGGCCCATTCTGACTAGGGGTGGAA
TCGAGCGGCTCGACTTGGCTCGTGAAATCTTGGCAAAGAATAGGCTTTAATGTAC
TGAATTAACGCCGAATTAATTCGGGGGATCTGGATTTTAGTACTGGATT

>NODE_5_length_397_cov_26.973684
TTTACACTTTATGCTTCCGGCTCGTATGTTGTGTGGAATTGTGAGCGGATAACAAT
TTCACACAGGAAACAGCTATGACATGATTACGAATTCGCCCTGGATTTTGGTTTT
AGGAATTAGAAATTTTATTGATAGAAGTATTTTACAAATACAAATACATACTAAG
TTGTACAAAAACCAGCAACTCACTGCACTGCACTTCACTTCACTTCACTGTATGA
ATAAAAGTCTGGTGTCTGGTTCCTGATCGATGACTGACTACTCCACTTTGTGCAG
AACAGATCTAGAGCTCGAGCTATTACTTGCGGAGGAACTCGCAGGCCTCGAGGA
GCGGGGAGGTGGAGCACTTCTTGAGGTTGGTGATGCCGGCCACGTACTGCGGGC
CGAGGTACTTCTC

>NODE_6_length_279_cov_4.107143
ATCATACAAACCTTGGATGATGTAGACCATGCTGAGAGTGTTGGTGTATCGAGGT
ACCAAAAGACCTTGAGGCTGAATGATACGTCGGATGACAAACGTACCAGTGTGC
TGGAATAATTCATTCCTCTCATCAACATACTCAGTCACACCACCTTCTGATCTCAC
TTTCTGAAGTGACTCAAATGCTTGTAATCTATCAAATCTATACTCACTAAAACTTC
CTTGATGAGGACTATGCCATGGGTTTGTTCTTGGATAAAATAGTTGGGCCATAGA
ATC

>NODE_7_length_266_cov_4.876777
GTATTCCTTGGTATCAGTCGTACCTTACGTCCCTTAAATACCGCGGTCGGGTATAA
CCCGTCAACAATGATCTCTCCTCTTTGGTCATTTTGACTCTGCGGCCTCTTTGCTG
CTACTGTGTTGATGCCTAAAGCCTCACTTAGTTGCTCAACACCGATTCTGTTGAAT
ATATTTTGCCTAGGGTGTTGCTCAATTGAGCTACCATATACTTGTTGAACTCTATT
GTTGTTAGCAGCTAATAAGAATTCCTGAAACTTTTATCAATG

>NODE_8_length_96_cov_8.658537
CCCAACAGTTGCGCAGCCTGAATGGCGAATGCTAGAGCAGCTTGAGCTTGGATCA
GATTGTCGTTTCCCGCCTTCAGTTTAAACTATCAGTGTTTG

Supplementary Sequence File 2. Assembled contigs from the read pairs mapping to the plasmid DNA sequence

>NODE_1_length_9145_cov_25.624972 (The sequence of one copy inserted T-DNA fragment)
CAAACACTGATAGTTTAAACTGAAGGCGGGAAACGACAATCTGATCCAAGCTCAAGCT
GCTCTAGCATTCGCCATTCAGGCTGCGCAACTGTTGGGAAGGGCGATCGGTGCGGGCC
TCTTCGCTATTACGCCAGCTGGCGAAAGGGGGATGTGCTGCAAGGCGATTAAGTTGGG
TAACGCCAGGGTTTTCCCAGTCACGACGTTGTAAAACGACGGCCAGTGCCAAGCTTTT
GGAAAGGTGCCGTGCAGTTCAAACAATTAGTTAGCAGTAGGGTGTTGGTTTTTGCTCA
CAGCAATAAGAAGTTAATCATGGTGTAGGCAACCCAAATAAAACACCAAAATATGCAC
AAGGCAGTTTGTTGTATTCTGTAGTACAGACAAAACTAAAAGTAATGAAAGAAGATGT
GGTGTTAGAAAAGGAAACAATATCATGAGTAATGTGTGAGCATTATGGGACCACGAAA
TAAAAAGAACATTTTGATGAGTCGTGTATCCTCGATGAGCCTCAAAAGTTCTCTCACCC
CGGATAAGAAACCCTTAAGCAATGTGCAAAGTTTGCATTCTCCACTGACATAATGCAAA
ATAAGATATCATCGATGACATAGCAACTCATGCATCATATCATGCCTCTCTCAACCTATTC
ATTCCTACTCATCTACATAAGTATCTTCAGCTAAATGTTAGAACATAAACCCATAAGTCA
CGTTTGATGAGTATTAGGCGTGACACATGACAAATCACAGACTCAAGCAAGATAAAGC
AAAATGATGTGTACATAAAACTCCAGAGCTATATGTCATATTGCAAAAAGAGGAGAGCT
TATAAGACAAGGCATGACTCACAAAAATTCATTTGCCTTTCGTGTCAAAAAGAGGAGG
GCTTTACATTATCCATGTCATATTGCAAAAGAAAGAGAGAAAGAACAACACAATGCTG
CGTCAATTATACATATCTGTATGTCCATCATTATTCATCCACCTTTCGTGTACCACACTTC
ATATATCATGAGTCACTTCATGTCTGGACATTAACAAACTCTATCTTAACATTTAGATGC
AAGAGCCTTTATCCCACTATAAATGCACGATGATTTCTCATTGTTTCTCACAAAAAGCAT
TCAGTTCATTAGTCCTACAACAACATGGCATCCATAAATCGCCCCATAGTTTTCTTCACA
GTTTGCTTGTTCCTCTTGTGCGATGGCTCCCTAGCCCAGTCTAGACTGGCTGGCCGCCG
CCGCCGCTCCGTGCAGTGGTGCACCGTGTCCCAGCCGGAGGCCACCAAGTGCTTCCA
GTGGCAGCGCAACATGCGCCGCGTGCGCGGCCCGCCGGTGTCCTGCATCAAGCGCGA
CTCCCCGATCCAGTGCATCCAGGCCATCGCCGAGAACCGCGCCGACGCCGTGACCCTC
GACGGCGGCTTCATCTACGAGGCCGGCCTCGCCCCGTACAAGCTCCGCCCGGTGGCCG
CCGAGGTGTACGGCACCGAGCGCCAGCCGCGCACCCACTACTACGCCGTGGCCGTGG
TGAAGAAGGGCGGCTCCTTCCAGCTCAACGAGCTGCAGGGCCTCAAGTCCTGCCACA
CCGGCCTCCGCCGCACCGCCGGCTGGAACGTGCCGATCGGCACCCTCCGCCCGTTCCT
CAACTGGACCGGCCCGCCGGAGCCGATCGAGGCCGCCGTGGCCCGCTTCTTCTCCGCC
TCCTGCGTGCCGGGCGCCGACAAGGGCCAGTTCCCGAACCTCTGCCGCCTCTGCGCCG
GCACCGGCGAGAACAAGTGCGCCTTCTCCTCCCAGGAGCCGTACTTCTCCTACTCCGG
CGCCTTCAAGTGCCTCCGCGACGGCGCCGGCGACGTGGCCTTCATCCGCGAGTCCACC
GTGTTCGAGGACCTCTCCGACGAGGCCGAGCGCGACGAGTACGAGCTGCTCTGCCCG
GACAACACCCGCAAGCCGGTGGACAAGTTCAAGGACTGCCACCTCGCCCGCGTGCCG
TCCCACGCCGTGGTGGCCCGCTCCGTGAACGGCAAGGAGGACGCCATCTGGAACCTC
CTCCGCCAGGCCCAGGAGAAGTTCGGCAAGGACAAGTCCCCGAAGTTCCAGCTCTTC
GGCTCCCCGTCCGGCCAGAAGGACCTCCTCTTCAAGGACTCCGCCATCGGCTTCTCCC
GCGTGCCGCCGCGCATCGACTCCGGCCTCTACCTCGGCTCCGGCTACTTCACCGCCATC
CAGAACCTCCGCAAGTCCGAGGAGGAGGTGGCCGCCCGCCGCGCCCGCGTGGTGTGG
TGCGCCGTGGGCGAGCAGGAGCTGCGCAAGTGCAACCAGTGGTCCGGCCTCTCCGAG

GGCTCCGTGACCTGCTCCTCCGCCTCCACCACCGAGGACTGCATCGCCCTCGTGCTCA
AGGGCGAGGCCGACGCCATGTCCCTCGACGGCGGCTACGTGTACACCGCCGGCAAGT
GCGGCCTCGTGCCGGTGCTCGCCGAGAACTACAAGTCCCAGCAGTCCTCCGACCCGG
ACCCGAACTGCGTGGACCGCCCGGTGGAGGGCTACCTCGCCGTGGCCGTGGTGCGCC
GCTCCGACACCTCCCTCACCTGGAACTCCGTGAAGGGCAAGAAGTCCTGCCACACCG
CCGTGGACCGCACCGCCGGCTGGAACATCCCGATGGGCCTCCTCTTCAACCAGACCGG
CTCCTGCAAGTTCGACGAGTACTTCTCCCAGTCCTGCGCCCCGGGCTCCGACCCGCGC
TCCAACCTCTGCGCCCTCTGCATCGGCGACGAGCAGGGCGAGAACAAGTGCGTGCCG
AACTCCAACGAGCGCTACTACGGCTACACCGGCGCCTTCCGCTGCCTCGCCGAGAACG
CCGGCGACGTGGCCTTCGTGAAGGACGTGACCGTGCTCCAGAACACCGACGGCAACA
ACAACGACGCCTGGGCCAAGGACCTCAAGCTCGCCGACTTCGCCCTCCTCTGCCTCGA
CGGCAAGCGCAAGCCGGTGACCGAGGCCCGCTCCTGCCACCTCGCCATGGCCCCGAA
CCACGCCGTGGTGTCCCGCATGGACAAGGTGGAGCGCCTCAAGCAGGTGCTCCTCCA
CCAGCAGGCCAAGTTCGGCCGCAACGGCTCCGACTGCCCGGACAAGTTCTGCCTCTTC
CAGTCCGAGACCAAGAACCTCCTCTTCAACGACAACACCGAGTGCCTCGCCCGCCTC
CACGGCAAGACCACCTACGAGAAGTACCTCGGCCCGCAGTACGTGGCCGGCATCACC
AACCTCAAGAAGTGCTCCACCTCCCCGCTCCTCGAGGCCTGCGAGTTCCTCCGCAAGT
AATAGCTCGAGCTCTAGATCTGTTCTGCACAAAGTGGAGTAGTCAGTCATCGATCAGG
AACCAGACACCAGACTTTTATTCATACAGTGAAGTGAAGTGAAGTGCAGTGCAGTGAG
TTGCTGGTTTTTGTACAACTTAGTATGTATTTGTATTTGTAAAATACTTCTATCAATAAAA
TTTCTAATTCCTAAAACCAAAATCCAGGGGTACCGAGCTTGCATGCCTGCAGTGCAGC
GTGACCCGGTCGTGCCCCTCTCTAGAGATAATGAGCATTGCATGTCTAAGTTATAAAAA
ATTACCACATATTTTTTTTGTCACACTTGTTTGAAGTGCAGTTTATCTATCTTTATACATAT
ATTTAAACTTTACTCTACGAATAATATAATCTATAGTACTACAATAATATCAGTGTTTTAG
AGAATCATATAAATGAACAGTTAGACATGGTCTAAAGGACAATTGAGTATTTTGACAAC
AGGACTCTACAGTTTTATCTTTTTAGTGTGCATGTGTTCTCCTTTTTTTTTGCAAATAGCT
TCACCTATATAATACTTCATCCATTTTATTAGTACATCCATTTAGGGTTTAGGGTTAATGGT
TTTTATAGACTAATTTTTTTAGTACATCTATTTTATTCTATTTTAGCCTCTAAATTAAGAAA
ACTAAAACTCTATTTTAGTTTTTTTATTTAATAATTTAGATATAAAATAGAATAAAATAAA
GTGACTAAAAATTAAACAAATACCCTTTAAGAAATTAAAAAAACTAAGGAAACATTTTT
CTTGTTTCGAGTAGATAATGCCAGCCTGTTAAACGCCGTCGACGAGTCTAACGGACAC
CAACCAGCGAACCAGCAGCGTCGCGTCGGGCCAAGCGAAGCAGACGGCACGGCATCT
CTGTCGCTGCCTCTGGACCCCTCTCGAGAGTTCCGCTCCACCGTTGGACTTGCTCCGCT
GTCGGCATCCAGAAATTGCGTGGCGGAGCGGCAGACGTGAGCCGGCACGGCAGGCGG
CCTCCTCCTCCTCTCACGGCACCGGCAGCTACGGGGGATTCCTTTCCCACCGCTCCTTC
GCTTTCCCTTCCTCGCCCGCCGTAATAAATAGACACCCCCTCCACACCCTCTTTCCCCA
ACCTCGTGTTGTTCGGAGCGCACACACACACACAACCAGATCTCCCCCAAATCCACCCGG
CGGCCCCTCCGCTTCAAGGTACGCCTCTCGTCCTCCCCCCCCCCTCTCTACCTTCTCTA
GATCGGCGTTCCGGTCCATGGTTAGGGCCCGGTAGTTCTACTTCTGTTCATGTTTGTGTT
AGATCCGTGTTTGTGTTAGATCCGTGCTGCTAGCGTTCGTACACGGATGCGACCTGTAC
GTCAGACACGTTCTGATTGCTAACTTGCCAGTGTTTCTCTTTGGGGAATCCTGGGATGG
CTCTAGCCGTTCCGCAGACGGGATCGATTTCATGATTTTTTTTGTTTCGTTGCATAGGGT
TTGGTTTGCCCTTTTCCTTTATTTCAATATATGCCGTGCACTTGTTTGTCGGGTCATCTTT
TCATGCTTTTTTTTGTCTTGGTTGTGATGATGTGGTCTGGTTGGGCGGTCGTTCTAGATC

GGAGTAGAATTCTGTTTCAAACTACCTGGTGGATTTATTAATTTTGGATCTGTATGTGTG
TGCCATACATATTCATAGTTACGAATTGAAGATGATGGATGGAAATATCGATCTAGGATA
GGTATACATGTTGATGCGGGTTTTACTGATGCATATACAGAGATGCTTTTTGTTCGCTTG
GTTGTGATGATGTGGTGTGGTTGGGCGGTCGTTCATTCGTTCTAGATCGGAGTAGAATA
CTGTTTCAAACTACCTGGTGTATTTATTAATTTTGGAACTGTATGTGTGTGTCATACATCT
TCATAGTTACGAGTTTAAGATGGATGGAAATATCGATCTAGGATAGGTATACATGTTGAT
GTGGGTTTTACTGATGCATATACATGATGGCATATGCAGCATCTATTCATATGCTCTAACC
TTGAGTACCTATCTATTATAATAAACAAGTATGTTTTATAATTATTTTGATCTTGATATACT
TGGATGATGGCATATGCAGCAGCTATATGTGGATTTTTTTAGCCCTGCCTTCATACGCTAT
TTATTTGCTTGGTACTGTTTCTTTTGTCGATGCTCACCCTGTTGTTTGGTGTTACTTCTGC
AGGTCGACTCTAGAGGATCTACCATGGCCACCGCCGCCGCCGCGTCTACCGCGCTCAC
TGGCGCCACTACCGCTGCGCCCAAGGCGAGGCGCCGGGCGCACCTCCTGGCCACCCG
CCGCGCCCTCGCCGCGCCCATCAGGTGCTCAGCGGCGTCACCCGCCATGCCGATGGCT
CCCCCGGCCACCCCGCTCCGGCCGTGGGGCCCCACCGATCCCCGCAAGGGATCTGGG
GCCAACGACCTGATCTTCCTGGCCCAGCCGGGCGGCAGGCTGAACGGCAGGATCAGG
GTGCCGGGCGACAAGAGCATCAGCCACAGGAGCATCATGCTGGGCAGCCTGGCCGAG
GGCACCACCGAGGTGGAGGGCTTCCTGGAGGGCGAGGACGCCCTGGCCACCCTGCAG
GCCTTCAGGGACATGGGCGTGGTGATCGAGGGCCCGAACCACGGCAGGGTGACCATC
CACGGCGTGGGCCTGCACGGCCTGAAGCCGCCGCCGGGCCCGCTGTACGTGGGCAAC
AGCGGCACCAGCATGAGGCTGCTGAGCGGCCTGCTGGCCGGCCAGAGCTTCGACGTG
ACCATGACCGGCGACGCCAGCCTGAGCAAGAGGCCGATGAACAGGGTGGCCAACCCG
CTGAGGGAGATGGGCGCCGTGGTGGAGACCGGCCCGGAGGGCAGGCCGCCGCTGAC
CATCAGGGGCGGCCACAAGCTGAAGGGCCTGACCTACACCCTGCCGATGGCCAGCGC
CCAGGTGAAGAGCTGCCTGCTGCTGGCCGGCCTGTACGCCGAGGGCAAGACCACCGT
GACCGAGCCGGCCCCGACCAGGGACCACACCGAGAGGATGCTGAGGGGCTTCGGCTA
CAGCGTGGAGAGCAACGGCCCGGTGGCCAGCCTGCAGAGCGGCGGCAAGCTGACCG
CCACCAGGATCGAGGTGCCGGCCGACATCAGCAGCGCCGCCTTCTTCCTGGTGGCCGC
CAGCATCGCCGAGGGCAGCGAGCTGGTGCTGGAGCACGTGGGCATCAACCCGACCAG
GACCGGCGTGATCGACATCCTGAGGCTGATGGGCGGCGACATCACCCTGGAGAACCA
GAGGGAGGTGGGCGGCGAGCCGGTGGCCGACCTGAGGGTGAGGGGCGCCCAGCTGA
AGGGCATCGACATCCCGGAGGCCCTGGTGCCGCTGGCCATCGACGAGTTCCCGGTGCT
GTTCGTGGCCGCCGCCTGCGCCGAGGGCAGGACCGTGCTGAGGGGCGCCGAGGAGCT
GAGGGTGAAGGAGAGCGACAGGATTCAGGTGATGGCCGACGGCCTGATCACCCTGGG
CATCAAGTGCGAGCCGACCCCGGACGGCATCATCATCGACGGCGGCCAGCTGGGCGG
CGGCGAGGTGCACGGCCACGGCGACCACAGGATCGCGATGGCCTTCAGCGTGGCCAG
CCTGAGGGCCAGCGCCCCGATCAGAATCCACGACTGCGCCAACGTGGCCACCAGCTT
CCCGAACTTCCTGGCCCTGTGCGCCGAGGTGGGCATCAGGGTGGCCGAGGAGGGCAA
GAGCTGACTCGAGCTCTAGATCTGTTCTGCACAAAGTGGAGTAGTCAGTCATCGATCA
GGAACCAGACACCAGACTTTTATTCATACAGTGAAGTGAAGTGAAGTGCAGTGCAGTG
AGTTGCTGGTTTTTGTACAACTTAGTATGTATTTGTATTTGTAAAATACTTCTATCAATAA
AATTTCTAATTCCTAAAACCAAAATCCAGGGCGAATTCGTAATCATGTCATAGCTGTTTC
CTGTGTGAAATTGTTATCCGCTCACAATTCCACACAACATACGAGCCGGAAGCATAAAG
TGTAAAGCCTGGGGTGCCTAATGAGTGAGCTAACTCACATTAATTGCGTTGCGCTCACT
GCCCGCTTTCCAGTCGGGAAACCTGTCGTGCCAGCTGCATTAATGAATCGGCCAACGC

GCGGGGAGAGGCGGTTTGCGTATTGGCTAGAGCAGCTTGCCAACATGGTGGAGCACG
ACACTCTCGTCTACTCCAAGAATATCAAAGATACAGTCTCAGAAGACCAAAGGGCTAT
TGAGACTTTTCAACAAAGGGTAATATCGGGAAACCTCCTCGGATTCCATTGCCCAGCTA
TCTGTCACTTCATCAAAAGGACAGTAGAAAAGGAAGGTGGCACCTACAAATGCCATCA
TTGCGATAAAGGAAAGGCTATCGTTCAAGATGCCTCTGCCGACAGTGGTCCCAAAGAT
GGACCCCCACCCACGAGGAGCATCGTGGAAAAAGAAGACGTTCCAACCACGTCTTCA
AAGCAAGTGGATTGATGTGAACATGGTGGAGCACGACACTCTCGTCTACTCCAAGAAT
ATCAAAGATACAGTCTCAGAAGACCAAAGGGCTATTGAGACTTTTCAACAAAGGGTAA
TATCGGGAAACCTCCTCGGATTCCATTGCCCAGCTATCTGTCACTTCATCAAAAGGACA
GTAGAAAAGGAAGGTGGCACCTACAAATGCCATCATTGCGATAAAGGAAAGGCTATCG
TTCAAGATGCCTCTGCCGACAGTGGTCCCAAAGATGGACCCCCACCCACGAGGAGCAT
CGTGGAAAAAGAAGACGTTCCAACCACGTCTTCAAAGCAAGTGGATTGATGTGATATC
TCCACTGACGTAAGGGATGACGCACAATCCCACTATCCTTCGCAAGACCCTTCCTCTAT
ATAAGGAAGTTCATTTCATTTGGAGAGGACACGCTGAAATCACCAGTCTCTCTCTACAA
ATCTATCTCTCTCGAGTGGAGCAAGAAGAGGATAGCTACAGAGAGAATGGCAATAATG
TAGGCGTTATCCATGGCTAAGCTGAGCTCACGAATGTGTGATGTGTTTCTGTGACTCTG
GTGCACTGCTAAGACTGGAGATCTGGATCCAGATCTCCAGTCTTGATCCAGGGAGAGG
TACTATGGGCTTAACTTTCCTCGGTTGCCCAGCAACTTACCAACAACAATTCCAACAAT
TTTCGCCTCAATGGCAAAGTGAAAGCCAAAAGTTTAGGGGCGAGCACCAACTGCAGG
GTTCAACCTAATTAGCACGGTTGTTTATGTCATAAACATATATGGTAACAACAGGTGCAT
CACCATCATTGTAGAACCAATGTGCAACACCAGCTGGGAGTGGAATAATATCTCCTTGT
CTAAATTGATAGATCTTTTGGTGCTCGTCCCTAAACTTTTGGCTTTCACTTTGCCATTGA
GGCGAAAATTGTTGGAATTGTTGTTGGTAAGTTGCTGGGCAACCGAGGAAAGTTAAGC
CCATAGTACCTCTCCCTGGATCAAGACTGGAGATCTGGATCCAGATCTCCAGTCTTAGC
AGTGCACCAGAGTCACAGAAACACATCACACATTCGTGAGCTCAGCTTAGCCATGGAT
AACGCCTACATTATTGCCATTCTCTCTGTAGCTATCCTCTTCTTGCTCCACTCGAGTTTCT
CCATAATAATGTGTGAGTAGTTCCCAGATAAGGGAATTAGGGTTCCTATAGGGTTTCGCT
CATGTGTTGAGCATATAAGAAACCCTTAGTATGTATTTGTATTTGTAAAATACTTCTATCA
ATAAAATTTCTAATTCCTAAAACCAAAATCCAGGG

>NODE_2_length_279_cov_4.107143
ATCATACAAACCTTGGATGATGTAGACCATGCTGAGAGTGTTGGTGTATCGAGGTACCA
AAAGACCTTGAGGCTGAATGATACGTCGGATGACAAACGTACCAGTGTGCTGGAATAA
TTCATTCCTCTCATCAACATACTCAGTCACACCACCTTCTGATCTCACTTTCTGAAGTGA
CTCAAATGCTTGTAATCTATCAAATCTATACTCACTAAAACTTCCTTGATGAGGACTATG
CCATGGGTTTGTTCTTGGATAAAATAGTTGGGCCATAGAATC

>NODE_3_length_266_cov_4.876777
GTATTCCTTGGTATCAGTCGTACCTTACGTCCCTTAAATACCGCGGTCGGGTATAACCCG
TCAACAATGATCTCTCCTCTTTGGTCATTTTGACTCTGCGGCCTCTTTGCTGCTACTGTG
TTGATGCCTAAAGCCTCACTTAGTTGCTCAACACCGATTCTGTTGAATATATTTTGCCTA
GGGTGTTGCTCAATTGAGCTACCATATACTTGTTGAACTCTATTGTTGTTAGCAGCTAAT
AAGAATTCCTGAAACTTTTATCAATG

>NODE_4_length_213_cov_3.765823

CGACTTATCAGCAACAATTCCAACAATTTTCGTCTCAAGGCCAAAGTCAAAGCCAAAA

GTTTAGGGATGAGCATCAAAAGATCCATCAATTTAGACAAGGAGATGTTGTTGCACTCC

CAGCTGGTGTTGCACATTGGTTCTACAATGATGGTGATGCATCGGTTGTTGCCATATATG

TTTATGACATAAACAACAGTGCAAATCAACTTGAAC

Supplementary Sequence File 3. The whole DNA sequence of mimic Gt1 gene insertion in artificial cisgenesis rice line.

1 CACCCAGTTG CCGCCGATTT CCCCGGAGCT GACGGCGCTG CTCGCCCGCC TCATGCTGGG

61 CTCCGGCGGC CACACCGGTC CTGAACCTGC TGGCTCGACG CCTCCGCCGA ACGCCCCCGT

121 CCTTGACGAC GCTACACGTG CTTCCCTCCA CGCTCAAGCC GTCGCTGTGC TCAACGTCAA

181 GGCCTTGGTA CCAGTGACTC TCGACCTCGC CGCCGGCAAC TACGCCCGCT GGCGCGGTCT

241 TTTCCTTGTG GTTCTCGGCA AATATGCTTT GACGGACCAC GTGCTTTGCG ACGCTCCTCG

301 CCCCGATCTC GCTGAGTGGG TGCAGATGGA CTGCGTCGTG CTCGGTTGGT TGTACGGTGC

361 CATCTCACCA GACCTTCTCC AAGAAGTTCT GTCGCCGACC GCCACCGCTC GCAGTGTTTG

421 GCGCGACCTG GAGTTCCAAT TCCTTGGAAA TTGTGAGCTC CGTGCTGTCA ATCTCTCCGC

481 CGAATTCCAC GGCTTCCAAC AAGGCGACCT CTCTGTCTCC GAGTATTGCC GGCGCCTCAA

541 GACGATGGTG GATAACCTCG CCGACCTCGG CGAACCATAA TCTGACCGCA CCCTCGTCCT

601 CACGCTTATC AACGGTCTCA GCCCTAAATT TGGCCACATG CAGTCTCTCC TTCCTATGCA

661 GCAGCCCTTT CCTAGTTTTA TTCAAGCCAG GTCTCAGCTG CTACTCGAGG AGATTACCAA

721 GGGACCACGT CCGGCGAACG ACTCGGCCAC CACTTTTGTT GCTACCACGG CCGGCGCCGG

781 CGGCACCCCC AGCGATCGTG GTGGCACCGG CAACACAGGC GGCTCCCAAG GTGGTTCTGG

841 CAGCGCCAGC GGCAACTCTC GCAACCGCCG ACGTGGCGGA CGCGGCAATG GTGGCGGCGG

901 CGGCAACACC AGCAATAGCG TCGCTGCCAA TCCTTCCGGT GGCTCGGGCG GTGCTTCTGA

961 TGGCCAATCA GCTGGCTCCC AGTCGGCCCA GCCCACTACT CGGCCGCCCT GGCCATCTCC

1021 CTACAACCCG GGTTGTCCAT TCTCTGTTTT CTCATATTGA TGAATCTCCA AGAGGATACC

1081 AACAGTACAT TGGAACGGGA ACACAAAAAT AGAAAGACCA TCTTGAGGGA TGTCCTTAGC

1141 ATTCAGAACT GATATTATAA GAAACTTTTA CAGTACATAG CAGCAACGCA ATTGCTTGCA

1201 ATTTATTTAT TTATTTATTT AGTTATTCTT TATCCGCATT GCCAACTTAA GAGGATTCCG

1261 CCACATTATA AACGTCTTGG TAGCTCTTGT ATTGGAGGGG AGTGAATGCA CCGAACTCAT

1321 CTCCTCTGTT ATGCTTGAGC CTCTGAGCCT CTTCTCTTGA GATGCGATAT GCATTTGCTA

1381 GAACATCAGT TGGGAGAGCA CGGAAGATGG AACTCTTTCC TGCAATGTGG CTTACCATAG

1441 AGTTAGGGTT TGTCTTGAAT GCAATGTAAG CACATCCTTC TCTTTGTGCC TTCTTTACAA

1501 CTACATAGTG TTGTGGTACA ATAAGTAGCT GTCCACGACG AAGCTCTCCG TTGAACACCG

1561 TCTTTCCATT GTTGTTGACA ACTTGAACCT GGGCTCGGCC TTGAGTAATA TACACGATGC

1621 TGTGAGCGTT GATGTTCCAG AACGGTGAAA GGAGTGCATT CTGCAATAGA ACAGATACAC

1681 ATCATTAAGC ATATTGTGTG CATTAAAGCT TTTGCCAAGA AAAAAAATGT GCTGTTCATT

1741 TACCTGGTAT AGATTTACTT TAACGGCGCT CATCTGTACA AGATTAAGAA TGGGGAAATT

1801 CTGGCTGTTG AGATTTGTAA CCCTTCCAGC TCTTGGGTTG TATGTATCAG CACGGTTAGG

1861 ATTATCGATG TTTTGCCTTA CCCTCATGGT GCAAAAGGTC TCATCCAAAC CGTTAGGGCA

1921 GCCACTCCCA TATTGACTTT GCTGATATCC TCCTTCTTGA TAATGCTCTC TTGATTGCAT

1981 TTGTCCTTGT TCCTGCTCTT GCAATGATGC ATATGGTTGC AGCAAACTGA GCCCGCGTTC

2041 AACGCGGACA ATTTCTCCTC TTTGGTCATT TTGACACTGG AGCTGCCTTG CAACTTGGTT

2101 GCTTATGCCA AAAGCCTCGC TAAGCAGTTC AGTGCTAAAG CCACTAAATA TGTTTTGTGA

2161 CCACTCCTCA ACTTCACGCC TGTATGCTTG AGGGTTTCTC TTATTTCCAG CTAACAAGAA

2221 ATCCTAACAA TTGATTACGA GGTTAAATCA TTGTCTAAGA AAAGTGGAAT AAAAAGGTCA

2281 AAATAATAGT AAAATGTTTA GTTTTGGATA AGTTTTGCTT AATTACCCTC TGTCGAGGGT

2341 CAAGTTGATT AGCACCGTTG TTGATATCAG TGACATATAT GGCAACAACC GGCACTTCAC

2401 CATCATTGTA GCACCAATGA GCTACACCAG CAGGCAACGC GATAACATCT CCTTGTCTGA

2461 AACGGTGAAT CTTTTGATGT TCATCCTTGA ACTTATGGCT TTGGCTTTGA CTTTCGGTCA

2521 ATTGGGCTTG CCCTGATTGT TGGAACTGCT GCTGGTAGGT CTCAGGACAG CCTGGGAAAG

2581 TCGGCCCTGT TATACCTCTC CCTGAAAGTA TGGCATGCAT GTTAATTACA AGCAATGTAA

2641 ATATCGCAGC CAATCATTAA TTCATTATGC ACTTAAATTG TTACACAAAC CTTGGATGAT

2701 ATATACTAGA GATGCACCAT TAGTGTAATG GGGTAGTAGT AGGCCTCTAG GTTCAATAAC

2761 TCGGCGGACA ACAGATACTC CGGTACATTG AAACAACTCA TTAGAGACAT CGAAGAACTC

2821 AGTTGTGCCA GCTTGAGACC TCACACTCCG AATTGGCTCA AATGCTTGCA ACCTATCAAA

2881 TCTACATCCT CTCGGACTTC CACGACGAGA ACTCTGCCAT TGACTAGTGC TCTGGCCTAA

2941 TAGCTGCTGG GCTAGGGAGC CATCGCACAA GAGGAACAAG CAAACTGTGA AGAAAACTAT

3001 GGGGCGATTT ATGGATGCCA TGTTGTTGTA GGACTAATGA ACTGAATGCT TTTTGTGAGA

3061 AACAATGAGA AATCATCGTG CATGGGCTGG CACTATTCAG ATGTGGCCTG GCCAGGCCCA

3121 CGGGCCAAAC TTCATGGGAA GGCCTCCTTT CGCGGGCGCT GCAGTGGCCG GACAGCCCAC

3181 CTACATGGTG CATCCTGCTT ACTATCCAGC TCACAACCAG GCCAGATTTC CTCAGCACAT

3241 GGGCTCGGCC GGTTTCCCTC AGTAGACCGG TCTCGCTCCC CTACAGCTAC CGGTGCCAAC

3301 TGCTTACAGC TCAGCCAGCT CTGTTCCTGC CACTCCGCAT GCATGGGCCG CTCCCTCACA

3361 GTGGGACCCG TAGGCCCTTG CCAGCTCCTT CAACACCTCC ACTTTGACGC CGCCAAACTC

3421 CAACGAGTGG TATATGGATT CAGGTGCATC TTCCCACATG ACTTCTAACA ATGGTACCCT

3481 TAGTGTGTCT CATCCTCCCC CCCCCCCCCC ACAATTTTCC TTCACATATT GTTGTTGGCA

3541 ATGGTTCACT AATTCCCATC ACCTCCACCG GCACCGCACA CTTACATTAT CCGCACCATT

3601 CATTTGTGCT TAATGATGTC TTAGTGTCAC CCTCTATTAT TAAAGATCTA ATCTCTGCCC

3661 GCCGGTTTGC TCGTGATAAT TCGTGTTCTG TTTGCCTTGA CCCTTTGGCC TTTCTGTGAA

3721 GGATTACCAA ACCAGGATCG AGATCGCCAG GTGCAATAGT TCCGGTGATT TGTACCCCCT

3781 CTCGGCGGAT TCCCTTCCAT CGGCAACCTC CGTACAAGCT TTTGTCGCCT CTACAACCAA

3841 CACCGACCTG TGGCATCGCC GCCTTGGACA TCTTGGTCAC GAGGCGCTCA CCCGCTTAGC

3901 TCAGGCTTCC GTCATCCCTC CTCCAAAAGG CGTTACCTCG TTGTGTCATG CTTGCCAACT

3961 CGGCCGTCAC ACCCGTCTTC CTTTTACTAG CATTTCACTA GGGCTACTGC CAAATTTGAG

4021 CTTATTCATT GTGATTTGTG GACCTCACCT ATACCAAGTG TGTCTGGTTT TAAATACTAT

4081 TTGGTCATTC TAGATGATTT CTCTCATTAC GTTTGGACCT TT

Supplementary Sequence File 4. Assembled contigs from 116 candidate read pairs artificial cisgenesis rice line.

>NODE_1_length_429_cov_3.96023
TAATTCATTATGCACTTAAATTGTTACACAAACCTTGGATGATATATACTAGAGATGCAC
CATTAGTGTAATGGGGTAGTAGTAGGCCTCTAGGTTCAATAACTCGGCGGACAACAGAT
ACTCCGGTACATTGAAACAACTCATTAGAGACATCGAAGAACTCAGTTGTGCCAGCTT
GAGACCTCACACTCCGAATTGGCTCAAATGCTTGCAACCTATCAAATCTACATCCTCTC
GGACTTCCACGACGAGAACTCTGCCATTGACTAGTGCTCTGGCCTAATAGCTGCTGGG
CTAGGGAGCCATCGCACAAGAGGAACAAGCAAACTGTGAAGAAAACTATGGGGCGAT
TTATGGATGCCATGTTGTTGTAGGACTAATGAACTGAATGCTTTTTGTGAGAAACAATG
AGAAATCATCGTGCATGGG

>NODE_2_length_408_cov_3.74018
ACCCGGGTTGTAGGGAGATGGCCAGGGCGGCCGAGTAGTGGGCTGGGCCGACTGGGA
GCCAGCTGATTGGCCATCAGAAGCACCGCCCGAGCCACCGGAAGGATTGGCAGCGAC
GCTATTGCTGGTGTTGCCGCCGCCGCCACCATTGCCGCGTCCGCCACGTCGGCGGTTG
CGAGAGTTGCCGCTGGCGCTGCCAGAACCACCTTGGGAGCCGCCTGTGTTGCCGGTG
CCACCACGATCGCTGGGGGTGCCGCCGGCGCCGGCCGTGGTAGCAACAAAAGTGGTG
GCCGAGTCGTTCGCCGGACGTGGTCCCTTGGTAATCTCCTCGAGTAGCAGCTGAGACC
TGGCTTGAATAAAACTAGGAAAGGGCTGCTGCATAGGAAGGAGAGACTGCATGTGGC
CAAATTT

>NODE_3_length_406_cov_4.26444
GCATGGGCTGGCACTATTCAGATGTGGCCTGGCCAGGCCCACGGGCCAAACTTCATGG
GAAGGCCTCCTTTCGCGGGCGCTGCAGTGGCCGGACAGCCCACCTACATGGTGCATCC
TGCTTACTATCCAGCTCACAACCAGGCCAGATTTCCTCAGCACATGGGCTCGGCCGGTT
TCCCTCAGTAGACCGGTCTCGCTCCCCTACAGCTACCGGTGCCAACTGCTTACAGCTCA
GCCAGCTCTGTTCCTGCCACTCCGCATGCATGGGCCGCTCCCTCACAGTGGGACCCGT
AGGCCCTTGCCAGCTCCTTCAACACCTCCACTTTGACGCCGCCAAACTCCAACGAGTG
GTATATGGATTCAGGTGCATCTTCCCACATGACTTCTAACAATGGTACCCTTAGTG

>NODE_4_length_408_cov_3.72205
ATGGTAAGCCACATTGCAGGAAAGAGTTCCATCTTCCGTGCTCTCCCAACTGATGTTCT
AGCAAATGCATATCGCATCTCAAGAGAAGAGGCTCAGAGGCTCAAGCATAACAGAGG
AGATGAGTTCGGTGCATTCACTCCCCTCCAATACAAGAGCTACCAAGACGTTTATAATG
TGGCGGAATCCTCTTAAGTTGGCAATGCGGATAAAGAATAACTAAATAAATAAATAAAT
AAATTGCAAGCAATTGCGTTGCTGCTATGTACTGTAAAAGTTTCTTATAATATCAGTTCT
GAATGCTAAGGACATCCCTCAAGATGGTCTTTCTATTTTTGTGTTCCCGTTCCAATGTAC
TGTTGGTATCCTCTTGGAGATTCATCAATATGAGAAAACAGAGAATGGACAACC
