# Supplementary material for: A paired-end whole-genome sequencing approach enables comprehensive characterization of transgene integration in rice
Source: Commun Biol. 2022 Jul 5;5:667. doi: 10.1038/s42003-022-03608-1 (PMC9256713; doi:10.1038/s42003-022-03608-1)
Supplement: Supplementary file 3 — Description of Additional Supplementary Files [file 42003_2022_3608_MOESM3_ESM.pdf]

Description of Additional Supplementary Files

File name: Supplementary Data 1
Description: The paired-end reads generated randomly based on the mimic Gt1 insertion using art-illumina software.
